# New score to predict COVID-19 progression in vaccine and early treatment era: the COVID-19 Sardinian Progression Score (CSPS)

**DOI:** 10.1186/s40001-024-01718-6

**Published:** 2024-02-15

**Authors:** Andrea De Vito, Laura Saderi, Agnese Colpani, Mariangela V. Puci, Beatrice Zauli, Vito Fiore, Marco Fois, Maria Chiara Meloni, Alessandra Bitti, Giulia Moi, Ivana Maida, Sergio Babudieri, Giovanni Sotgiu, Giordano Madeddu

**Affiliations:** 1 Unit of Infectious Disease, Department of Medicine, Surgery and Pharmacy, University of Sassari, 07100 Sassari, Italy; 2 PhD School in Biomedical Science, Biomedical Science Department, University of Sassari, Sassari, Italy; 3 Clinical Epidemiology and Medical Statistics Unit, Department of Medicine, Surgery and Pharmacy, University of Sassari, 07100 Sassari, Italy

**Keywords:** Progression risk score, COVID-19, Score, SARS-CoV-2, Antiviral treatment, Vaccination

## Abstract

**Background:**

Several scores aimed at predicting COVID-19 progression have been proposed. As the variables vaccination and early SARS-CoV-2 treatment were systematically excluded from the prognostic scores, the present study's objective was to develop a new model adapted to the current epidemiological scenario.

**Methods:**

We included all patients evaluated by the Infectious Disease Unit in Sassari, with SARS-CoV-2 infection and without signs of respiratory failure at the first evaluation (P/F > 300). Disease progression was defined by the prescription of supplemental oxygen. In addition, variables related to demographics, vaccines, comorbidities, symptoms, CT scans, blood tests, and therapies were collected. Multivariate logistic regression modelling was performed to determine factors associated with progression; any variable with significant univariate test or clinical relevance was selected as a candidate for multivariate analysis. Hosmer–Lemeshow (HL) goodness of fit statistic was calculated. Odds ratio values were used to derive an integer score for developing an easy-to-use progression risk score. The discrimination performance of the risk index was determined using the AUC, and the best cut-off point, according to the Youden index, sensitivity, specificity, predictive value, and likelihood ratio, was chosen.

**Results:**

1145 patients [median (IQR) age 74 (62–83) years; 53.5% males] were enrolled; 336 (29.3%) had disease progression. Patients with a clinical progression were older and showed more comorbidities; furthermore, they were less vaccinated and exposed to preventive therapy. In the multivariate logistic regression analysis, age ≥ 60 years, COPD, dementia, haematological tumours, heart failure, exposure to no or one vaccine dose, fever, dyspnoea, GGO, consolidation, ferritin, De Ritis ≥ 1.2, LDH, and no exposure to early anti-SARS-CoV-2 treatment were associated with disease progression. The final risk score ranged from 0 to 45. The ROC curve analysis showed an AUC of 0.92 (95% CI 0.90–0.93) with a 93.7% specificity and 72.9% sensitivity. Low risk was defined when the cut-off value was less than 23. Three risk levels were identified: low (0–23 points), medium (24–35), and high (≥ 36).

**Conclusions:**

The proportion of patients with progression increases with high scores: the assessment of the risk could be helpful for clinicians to plan appropriate therapeutic strategies.

## Introduction

More than six million deaths have occurred since the emergence and spread of SARS-CoV-2 worldwide; however, asymptomatic or mild forms of disease are recorded in the majority of the cases [1]. The most prevalent symptoms of Coronavirus Disease 19 (COVID-19) are fever, cough, and dyspnoea; a low proportion complains of gastrointestinal symptoms, anosmia, dysgeusia, headache, and skin lesions [2–4] life-threatening systemic inflammation, respiratory failure, and multiorgan dysfunction [5, 6].

Several factors are associated with COVID-19 severity and death: older age, being male, being a smoker, Chronic Obstructive Pulmonary Disease (COPD), cardiovascular disease (CVD), diabetes, hypertension, obesity, cancer, and acute kidney injury are associated with increased mortality [7].

Several scores were created to identify individuals with a higher risk of severe disease and death [6, 8–12]. The 4C-score, developed at the beginning of the pandemic after the recruitment of a cohort in the ISARIC Coronavirus Clinical Characterization Consortium (ICARIC-4C), has been frequently adopted [13]. However, in these years, substantial changes occurred. First, the virus mutated with new viral variants [14, 15]. In addition, several vaccines were commercially distributed, reducing the risk of severe illness and death [16, 17]. Finally, more effective drugs and adequate management of people with severe diseases [18–23]. For all these reasons, it is not easy for clinicians to predict the patients' evolution, given the numerous factors that come into play and decide when hospital admission could be necessary. In this regard, Drake et al. found that only half of the hospitalized people developed complications needing support [24].

Therefore, aim of the present study was to create a new score to predict the risk of disease severity.

## Methods

### Study design

A retrospective cohort study was conducted by recruiting individuals with a SARS-CoV-2 infection diagnosed by Polymerase Chain Reaction (PCR) between January and September 2022 in an Italian university hospital. Those aged < 18 years, those with incomplete clinical data, and with severe COVID-19 needing oxygen supplementation at the first evaluation were excluded.

The primary study objective was to create a score to predict disease progression (i.e., administration or increase of oxygen supplementation).

Data on demographics (age, gender, and weight), medical history (chronic renal disease, dialysis, immunodeficiency, transplantation, rheumatologic disease, diabetes, COPD, hemoglobinopathy, neurological disease, cancer, and cardiovascular disease), Charlson Comorbidity Index (CCI) [25], vaccination status (number of doses, time from the last dose), ward and symptoms at the admission (fever, cough, tachypnea, ageusia, pharyngodynia, chills, asthenia, headache, myalgia, gastrointestinal symptoms, dyspnoea, nasal congestion, and anosmia), computed tomography (CT) signs, biochemical indicators at admission (white blood cells -WBC-, neutrophils lymphocyte, neutrophil–lymphocyte-ratio-NLR-, ferritin, procalcitonin -PCT-, urea, creatinine, Estimated Glomerular Filtration Rate –eGFR—calculated with Cockcroft-Gault formula [26], aspartate aminotransferase -AST-, alanine aminotransferase -ALT-, De Ritis ratio -AST/ALT-, lactate dehydrogenase -LDH-, C-reactive protein -CRP-, and D-Dimer), early treatment with antivirals [monlupiravir, nirmatrelvir/ritonavir(r), and remdesivir] and monoclonal antibodies (casirivimab/imdevimab, and sotrovimab), and data of negativization were collected.

### Ethics

The study was conducted in accordance with the Declaration of Helsinki and approved by the Institutional Ethics Committee with the protocol code PG/2022/20481.

### Statistical analysis

Sample characteristics were described using absolute and relative (percentage) frequencies or median and Interquartile Range (IQR); Shapiro–Wilk test was used to assess the normality distribution of quantitative variables. Differences between quantitative and qualitative variables were evaluated by the Mann–Whitney *U* test and by Pearson Chi-Square or Fisher exact test, respectively. A multivariate stepwise logistic regression was performed to assess disease severity-related factors. The variables included in the multivariate model were evaluated based on their clinical or statistical significance at the obtained in the univariate analysis. Hosmer–Lemeshow (HL), goodness of fit statistic was calculated. Odds ratio values were used to derive an integer score from developing an easy-to-use progression risk score. The discrimination performance of the risk index was determined using the AUC, and the best cut-off, according to the Youden index, sensitivity, specificity, predictive value, and likelihood ratio, was chosen. Furthermore, performance of the score (AUC) was tested to a half cohort randomly selected from the total cohort. A two-tailed *p*-value less than 0.05 was considered statistically significant. Statistical analysis was carried out using STATA 17 (StataCorp, TX, USA).

## Results

A total of 1652 individuals with a SARS-CoV-2 infection were evaluated: 245 were excluded because of missing data, as well as 262 owing to severe COVID-19 at the admission.

Overall, 1145 patients with a median (IQR) age of 74 (62–83) years were included in the study. During the follow-up, 336 (29.3%) developed a severe disease needing oxygen supplementation or an increased administration. When compared with patients who did not experience disease progression, they were older, with a higher comorbidity index, less frequently vaccinated and exposed to early antiviral therapies and monoclonal antibodies, with fever, cough, and dyspnea at admission, and more frequently showed CT and blood biochemical indexes abnormalities (Table 1).

**Table 1:** Demographic characteristics, comorbidities, symptoms, radiological findings, biochemical features, and treatments of 1145 patients with SARS-CoV-2 infection with or without disease progression

Variables	Total cohort (*n* = 1145)	Non-severe disease (*n* = 809)	Severe disease (*n* = 336)	*p*-value
Males, *n* (%)	612 (53.5)	426 (52.7)	186 (55.4)	0.404
Age, years, median (IQR)	74 (62–83)	72 (59–82)	77.5 (66.5–86.0)	< 0.001
Age groups, *n* (%)				
< 50 years	134 (11.7)	118 (14.6)	16 (4.8)	< 0.001
50–59 years	115 (10.0)	90 (11.1)	25 (7.4)	0.058
60–69 years	209 (18.3)	147 (18.2)	62 (18.5)	0.905
70–79 years	270 (23.6)	182 (22.5)	88 (26.2)	0.179
≥ 80 years	417 (36.4)	272 (33.6)	145 (43.2)	0.002
Age ≥ 60 years, *n* (%)	896 (78.3)	601 (74.3)	295 (87.8)	< 0.001
Patient provenience, *n* (%)				
ED	762 (66.7)	508 (62.9)	254 (75.8)	< 0.001
Ward	334 (29.2)	255 (31.6)	79 (23.6)	0.007
Domicile	47 (4.1)	45 (5.6)	2 (0.6)	0.001
*Comorbidities*				
Weight, kg, median (IQR)	70 (60–80)	70 (60–80)	70 (62–80)	0.111
BMI > 30 kg/m^2^, *n* (%)	260 (22.7)	173 (21.4)	87 (25.9)	0.097
Chronic renal failure, *n* (%)	186 (16.2)	127 (15.7)	59 (17.6)	0.437
Dialysis, *n* (%)	24 (2.1)	15 (1.9)	9 (2.7)	0.375
Immunodeficit, *n* (%)	156 (13.6)	104 (12.9)	52 (15.5)	0.239
Transplant recipients, *n* (%)	17 (1.5)	10 (1.2)	7 (2.8)	0.280
Rheumatological disease, *n* (%)	62 (5.4)	42 (5.2)	20 (6.0)	0.604
Decompensated diabetes, *n* (%)	183 (16.0)	109 (13.5)	74 (22.0)	< 0.001
Diabetes, *n* (%)	252 (22.0)	172 (21.3)	80 (23.8)	0.343
Chronic liver disease, *n* (%)	66 (5.8)	42 (5.2)	24 (7.1)	0.197
COPD, *n* (%)	222 (19.4)	137 (16.9)	85 (25.3)	0.001
Hemoglobinopathies, *n* (%)	5 (0.4)	4 (0.5)	1 (0.3)	0.646
Neurodevelopmental/neurodegenerative diseases, *n* (%)	321 (28.0)	203 (25.1)	118 (35.1)	0.001
Dementia, *n* (%)	176 (15.4)	100 (12.4)	76 (22.6)	< 0.001
Chromosopathies/hypoxia, *n* (%)	8 (0.7)	4 (0.5)	4 (1.2)	0.198
Neuromuscular disease, *n* (%)	33 (2.9)	25 (3.1)	8 (2.4)	0.514
Cerebrovascular events, *n* (%)	134 (11.7)	88 (10.9)	46 (13.7)	0.178
Oncological disease, *n* (%)	170 (14.9)	133 (16.4)	37 (11.0)	0.019
Metastasis, *n* (%)	58 (5.1)	41 (5.1)	17 (5.1)	0.995
Terminal cancer, *n* (%)	20 (1.8)	6 (0.7)	14 (4.2)	< 0.001
Haematological tumours, *n* (%)	71 (6.2)	44 (5.4)	27 (8.0)	0.097
Solid tumours in chemotherapy, *n* (%)	33 (2.9)	26 (3.2)	7 (2.1)	0.298
Haematological tumours in chemotherapy, *n* (%)	48 (4.2)	30 (3.7)	18 (5.4)	0.205
Cardiovascular diseases, *n* (%)	417 (36.4)	276 (34.1)	141 (42.0)	0.012
Heart failure, *n* (%)	370 (32.3)	241 (29.8)	129 (38.4)	0.005
Previous acute myocardial infarction, *n* (%)	147 (12.8)	101 (12.5)	46 (13.7)	0.579
Hypertension, *n* (%)	547 (47.8)	361 (44.6)	186 (55.4)	0.001
Median (IQR) number of comorbidities	2 (1–3)	2 (1–3)	2 (1–3)	< 0.001
CCI, median (IQR)	5 (3–7)	5 (3–7)	5 (4–7)	< 0.001
Vaccine, *n* (%)	937 (81.8)	721 (89.1)	216 (64.3)	< 0.001
N. of doses, *n* (%)				
0	208 (18.2)	88 (10.9)	120 (35.7)	< 0.001
1	26 (2.3)	16 (2.0)	10 (3.0)	0.303
2	187 (16.3)	140 (17.3)	47 (14.0)	0.169
3	698 (61.0)	543 (67.1)	155 (46.1)	< 0.001
4	26 (2.3)	22 (2.7)	4 (1.2)	0.120
Time between vaccination and SARS-CoV-2 infection, median (IQR)	147 (84–204)	136 (82–191)	171.5 (98–227)	0.001
*Symptoms*^a^	954 (83.3)	639 (79.0)	315 (93.8)	< 0.001
Fever, *n* (%)	538 (47.0)	335 (41.4)	203 (60.4)	< 0.001
Cough, *n* (%)	410 (35.8)	257 (31.8)	153 (45.5)	< 0.001
Tachypnoea, *n* (%)	35 (3.1)	12 (1.5)	23 (6.9)	< 0.001
Ageusia, *n* (%)	17 (1.5)	13 (1.6)	4 (1.2)	0.596
Pharyngodynia, *n* (%)	162 (14.2)	132 (16.3)	30 (8.9)	0.001
Chills, *n* (%)	40 (3.5)	33 (4.1)	7 (2.1)	0.094
Asthenia, *n* (%)	410 (35.8)	290 (35.9)	120 (35.7)	0.996
Headache, *n* (%)	127 (11.1)	94 (11.6)	33 (9.8)	0.378
Myalgias, *n* (%)	184 (16.1)	134 (16.6)	50 (14.9)	0.480
Gastrointestinal symptoms, *n* (%)	171 (14.9)	120 (14.8)	51 (15.2)	0.881
Dyspnoea, *n* (%)	281 (24.5)	102 (12.6)	179 (53.3)	< 0.001
Nasal congestion, *n* (%)	53 (4.6)	49 (6.1)	4 (1.2)	< 0.001
Anosmia, *n* (%)	21 (1.8)	14 (1.7)	7 (2.1)	0.685
*Radiological findings*				
CT pneumonia, *n* (%)	449 (39.2)	193 (23.9)	256 (79.2)	< 0.0001
GGO, *n* (%)	375 (32.8)	157 (19.4)	218 (64.9)	< 0.0001
Consolidation, *n* (%)	227 (19.8)	79 (9.8)	148 (44.1)	< 0.0001
Pulmonary embolism, *n* (%)	21 (1.8)	12 (1.5)	9 (2.7)	0.170
*Biochemical indexes*				
WBC (× 10^3^), median (IQR)	6.9 (5.3–9.3)	6.7 (5.1–9.0)	7.6 (5.7–10.4)	0.001
Neutrophils, median (IQR)	4.9 (3.4–7.1)	4.8 (3.3–6.6)	5.7 (3.9–8.3)	< 0.001
Lymphocytes, median (IQR)	1.1 (0.7–1.6)	1.1 (0.8–1.6)	0.9 (0.6–1.4)	0.001
NLR, median (IQR)	4.5 (2.6–8.0)	4 (2.5–7.2)	5.8 (3.1–10.2)	< 0.001
Ferritin, median (IQR)	222 (121–454)	187 (107–349)	411.5 (199.5–874.0)	< 0.001
Ferritin/10, median (IQR)	22.2 (12.1–45.4)	18.7 (10.7–34.9)	41.2 (20.0–87.4)	< 0.001
Ferritin/50, median (IQR)	4.4 (2.4–9.1)	3.7 (2.1–7.0)	8.2 (4.0–17.5)	< 0.001
PCT, median (IQR)	0.07 (0.02–0.22)	0.05 (0.02–0.15)	0.15 (0.06–0.46)	< 0.001
PCT > 0.5, *n* (%)	174 (15.2)	93 (11.5)	81 (24.1)	
Urea, median (IQR)	35 (27–55)	33 (25–50)	44 (32–65)	< 0.001
Creatinine, median (IQR)	0.9 (0.7–1.3)	0.8 (0.7–1.2)	1.0 (0.8–1.3)	0.002
eGFR, median (IQR)	67.4 (43.6–90.7)	71.4 (45.8–92.7)	57.4 (37.9–82.2)	< 0.001
AST, median (IQR)	23 (17–33)	22 (17–30)	26.5 (19.0–40.5)	< 0.001
ALT, median (IQR)	19 (13–30)	18 (13–29)	20 (13–32)	0.212
De Ritis, median (IQR)	1.2 (0.9–1.7)	1.2 (0.9–1.5)	1.3 (1.1–1.8)	< 0.001
LDH, median (IQR)	222 (177–280)	206 (168–250)	262 (212.5–333.5)	< 0.001
LDH/10, median (IQR)	22.2 (17.7–28.0)	20.6 (16.8–25.0)	26.2 (21.3–33.4)	< 0.001
LDH/50, median (IQR)	4.4 (3.5–5.6)	4.2 (3.4–5.0)	5.2 (4.3–6.7)	< 0.001
CRP, median (IQR)	2.6 (1.1–6.6)	2.0 (1.0–5.2)	5.6 (2.3–11.1)	< 0.001
D-Dimer, median (IQR)	1 (0.5–2.1)	0.8 (0.4–1.8)	1.5 (0.8–3.3)	< 0.001
*Therapy*				
Days between symptoms onset and start of treatment, median (IQR)	2 (1–3)	2 (1–3)	2 (1–4)	0.154
Early treatment, *n* (%)	214 (65.6)	190 (66.7)	24 (58.5)	0.305
Antiviral, *n* (%)	389 (34.0)	352 (43.5)	37 (11.0)	< 0.001
Monlupiravir, *n* (%)	242 (21.1)	224 (27.7)	18 (5.4)	< 0.001
Nirmatrelvir/ritonavir, *n* (%)	39 (3.4)	36 (4.5)	3 (0.9)	0.002
Remdesivir, *n* (%)	108 (9.4)	92 (11.4)	16 (4.8)	< 0.001
Monoclonal antibodies, *n* (%)	237 (20.7)	207 (25.6)	30 (8.9)	< 0.001
Casirivimab/Imdevimab, *n* (%)	110 (9.6)	91(11.3)	19 (5.7)	0.003
Sotrovimab, *n* (%)	130 (11.4)	118 (14.6)	12 (3.6)	< 0.001
Hospital-acquired infection, *n* (%)	304 (26.6)	233 (28.8)	71 (21.1)	0.007
Bacterial co-infection, *n* (%)	129 (11.3)	71 (8.8)	58 (17.3)	< 0.001

^a^People with at least one symptom

Having more than 60 years, COPD, dementia, haematological tumor, heart failure, having not received at least two doses of vaccine, fever and dyspnea, CT ground glass opacities (GGO) and consolidation, higher ferritin and LDH level, De Ritis ratio, and not having received antivirals or monoclonal antibodies were associated with an increased risk of disease severity (Table 2).

**Table 2:** Logistic regression analysis to assess the relationship between demographic, clinical characteristics and the need to start oxygen therapy (*n* = 1145)

Variables	Univariate analysis		Multivariate analysis	
	OR (95% CI)	*p*-value	OR (95% CI)	*p*-value
Males	1.12 (0.86–1.44)	0.404	–	–
Age groups ≥ 60 years^a^	2.49 (1.73–3.58)	< 0.0001	2.86 (1.52–5.37)	0.001
*Comorbidity*				
Weight, kg	1.00 (0.99–1.01)	0.298	–	–
BMI > 30 kg/m^2^	1.28 (0.96–1.73)	0.098	–	–
Chronic renal failure, yes	1.14 (0.82–1.61)	0.437	–	–
Dialysis, yes	1.46 (0.63–3.36)	0.378	–	–
Immunodeficit, yes	1.24 (0.87–1.78)	0.240	–	–
Transplant recipients, yes	1.70 (0.64–4.50)	0.286	–	–
Rheumatological disease, yes	1.16 (0.67–2.00)	0.605	–	–
Decompensated diabetes, yes^a^	1.81 (1.31–2.52)	< 0.001	1.40 (0.84–2.32)	0.198
Diabetes, yes	1.16 (0.86–1.57)	0.343	–	–
Chronic liver disease, yes	1.41 (0.84–23.36)	0.199	–	–
COPD, yes^a^	1.66 (1.22–2.26)	0.001	1.69 (1.03–2.78)	0.038
Hemoglobinopathies, yes	0.60 (0.07–5.40)	0.649	–	–
Neurodevelopmental/neurodegenerative diseases, yes	1.62 (1.23–2.13)	0.001	–	–
Dementia, yes^a^	2.07 (1.49–2.88)	< 0.0001	2.17 (1.32–3.56)	0.002
Chromosopathies/hypoxia, yes	2.43 (0.60–9.75)	0.212	–	–
Neuromuscular disease, yes	0.77 (0.34–1.71)	0.515	–	–
Cerebrovascular events, yes	1.30 (0.89–1.90)	0.178	–	–
Oncological disease, yes^a^	0.63 (0.43–0.93)	0.019	0.93 (0.51–1.72)	0.822
Metastasis, yes	1.00 (0.56–1.78)	0.995	–	–
Terminal cancer, yes	5.82 (2.22–15.27)	< 0.0001	–	–
Hematological tumors, yes^a^	1.52 (0.92–2.50)	0.099	3.01 (1.41–6.543)	0.004
Solid tumors in chemotherapy, yes	0.64 (0.28–1.49)	0.302	–	–
Hematological tumors in chemotherapy, yes	1.47 (0.81–2.68)	0.207	–	–
Cardiovascular diseases, yes	1.40 (1.08–1.81)	0.012	–	–
Heart failure, yes^a^	1.47 (1.13–1.92)	0.005	1.55 (1.01–2.38)	0.046
Previous acute myocardial infarction, yes	1.11 (0.76–1.62)	0.579	–	–
Hypertension, yes	1.54 (1.19–1.99)	0.001	–	–
Number of comorbidities, yes	1.22 (1.12–1.34)	< 0.001	–	–
CCI, yes	1.11 (1.06–1.16)	< 0.001	–	–
Vaccine 0–1 doses^a^	4.28 (3.17–5.78)	< 0.001	3.46 (2.19–5.49)	< 0.001
*Symptoms*	3.99 (2.49–6.40)	< 0.001	–	–
Fever, yes^a^	2.16 (1.67–2.80)	< 0.001	2.60 (1.75–3.88)	< 0.001
Cough, yes	1.80 (1.39–2.33)	< 0.001	–	–
Tachypnea, yes	4.88 (2.40–9.93)	< 0.001	–	–
Ageusia, yes	0.74 (0.24–2.28)	0.597	–	–
Pharyngodynia, yes	0.50 (0.33–0.76)	0.001	–	–
Chills, yes	0.50 (0.22–1.14)	0.100	–	–
Asthenia, yes	0.99 (0.76–1.30)	0.966	–	–
Headache, yes	0.83 (0.55–1.26)	0.378	–	–
Myalgias, yes	0.88 (0.62–1.25)	0.480	–	–
Gastrointestinal symptoms, yes	1.03 (0.72–1.47)	0.881	–	–
Dyspnoea, yes^a^	7.90 (5.86–10.65)	< 0.001	5.04 (3.24–7.83)	< 0.001
Nasal congestion, yes	0.19 (0.07–0.52)	0.001	–	–
Anosmia, yes	1.21 (0.48–3.02)	0.686	–	–
*Radiological findings*			–	–
CT pneumonia, yes	10.21 (7.58–13.77)	< 0.0001	–	–
GGO, yes^a^	7.67 (5.78–10.19)	< 0.0001	3.52 (2.33–5.32)	< 0.001
Consolidation, yes^a^	7.27 (5.30–9.98)	< 0.0001	2.67 (1.66–4.29)	< 0.001
Pulmonary Embolism, yes	1.83 (0.76–4.38)	0.176	–	–
*Biochemical indexes*				
WBC (× 10^3^) ≥ 11^a^	2.10 (1.50–2.95)	< 0.0001	1.35 (0.79–2.31)	0.271
Neutrophils	1.01 (0.99–1.03)	0.161	–	–
Lymphocytes	1.04 (0.99–1.09)	0.115	–	–
NLR	1.01 (0.99–1.03)	0.095	–	–
Ferritin	1.00 (1.00–1.00)	< 0.001	–	–
Ferritin > 336 ng/mL in male and > 307 ng/mL in female^a^	3.75 (2.87–4.89)	< 0.001	3.24 (2.16–4.85)	< 0.001
PCT	1.01 (0.99–1.03)	0.317	–	–
PCT > 0.5 ng/mL^a^	2.45 (1.76–3.40)	< 0.001	1.03 (0.61–1.73)	0.914
Urea	1.01 (1.01–1.01)	< 0.001	–	–
Creatinine	1.01 (0.97–1.05)	0.713	–	–
eGFR ≥ 67.4 mL/min/1.73m^2a^	0.50 (0.38–0.65)	< 0.001	0.75 (0.49–1.15)	0.186
AST	1.00 (1.00–1.01)	0.004	–	–
ALT	1.00 (1.00–1.00)	0.093	–	–
De Ritis ≥ 1.2^a^	1.92 (1.48–2.50)	< 0.0001	1.60 (1.07–2.39)	0.022
LDH	1.00 (1.00–1.01)	< 0.001	–	–
LDH > 333 UI/L^a^	3.37 (2.39–4.74)	< 0.001	1.79 (1.01–3.18)	0.048
CRP	1.00 (0.99–1.00)	0.785	–	–
D-Dimer ≥ 1^a^	2.21 (1.70–2.88)	< 0.001	1.27 (0.86–1.90)	0.234
*Therapy*				
Early treatment, yes	0.71 (0.36–1.38)	0.307	–	–
Exposure to antiviral	0.16 (0.11–0.23)	< 0.001	–	–
Exposure to Monlupiravir, yes	0.15 (0.09–0.24)	< 0.001	–	–
Exposure to Nirmatrelvir/ritonavir	0.19 (0.06–0.63)	0.007	–	–
Exposure to Remdesivir	0.39 (0.23–0.67)	0.001	–	–
Not therapy with antiviral or monoclonal antibodies treatment^a^	7.13 (5.12–9.93)	< 0.001	11.07 (6.99–17.54)	< 0.001
Exposure to Casirivimab/Imdevimab	0.47 (0.28–0.79)	0.004	–	–
Exposure to Sotrovimab	0.22 (0.12–0.40)	< 0.001	–	–
Hospital infection, yes	0.66 (0.49–0.90)	0.008	–	–
Bacterial co-infection, yes^a^	2.17 (1.49–3.15)	< 0.001	1.55 (0.88–2.74)	0.131

Hosmer–Lemeshow *p*-value = 0.36

^a^Variables included in the multivariate analysis

A score was assigned according to the odd ratio (Table 3).

**Table 3:** Variables included for the score estimate

Variables	OR (95% CI)	Score
Age groups ≥ 60 years	2.86 (1.52–5.37)	3
Decompensated diabetes, yes	1.40 (0.84–2.32)	1
COPD/Emphysema, yes	1.69 (1.03–2.78)	2
Dementia, yes	2.17 (1.32–3.56)	2
Oncological disease, yes	0.93 (0.51–1.72)	1
Hematological tumors, yes	3.01 (1.41–6.543)	3
Heart failure, yes	1.55 (1.01–2.38)	2
Vaccine 0–1 doses, yes	3.46 (2.19–5.49)	3
Fever, yes	2.60 (1.75–3.88)	3
Dyspnoea, yes	5.04 (3.24–7.83)	5
GGO, yes	3.52 (2.33–5.32)	4
Consolidation	2.67 (1.66–4.29)	3
WBC (× 10^3) ≥ 11	1.35 (0.79–2.31)	1
Ferritin > 336 ng/mL in male and > 336 ng/mL in female	3.24 (2.16–4.85)	3
PCT > 0.5	1.03 (0.61–1.73)	1
eGFR ≥ 67.4	0.75 (0.49–1.15)	1
De Ritis ≥ 1.2	1.60 (1.07–2.39)	2
LDH > 333 Ui/L	1.79 (1.01–3.18)	2
D-Dimer ≥ 1	1.27 (0.86–1.90)	1
Not therapy with antiviral or monoclonal antibodies	11.07 (6.99–17.54)	11
Bacterial infection, yes	1.55 (0.88–2.74)	2

The risk score ranged from 0 to 45 points. According to the Youden Index and clinical considerations, a low risk of clinical progression was considered for values less than 23 (Table 4). Patients were divided into three different risk groups: Low (0–23 points), Medium (24–35 points), and High (≥ 36 points).

**Table 4:** Progression risk score description

	Total cohort (*n* = 1145)	Non-severe disease (*n* = 809)	Severe disease (*n* = 336)	*p*-value
Progression risk score, median (IQR)	19 (13–26)	15 (11–20)	30 (25–35)	< 0.001
Risk score cut-off > 23, *n* (%)	357 (31.2)	92 (11.4)	265 (78.9)	< 0.001
Risk score levels, *n* (%)				
Low	788 (88.8)	717 (88.6)	71 (21.1)	< 0.001
Medium	288 (25.2)	90 (11.1)	198 (58.9)	
High	69 (6.0)	2 (0.3)	67 (19.9)	

**Fig. 1:**
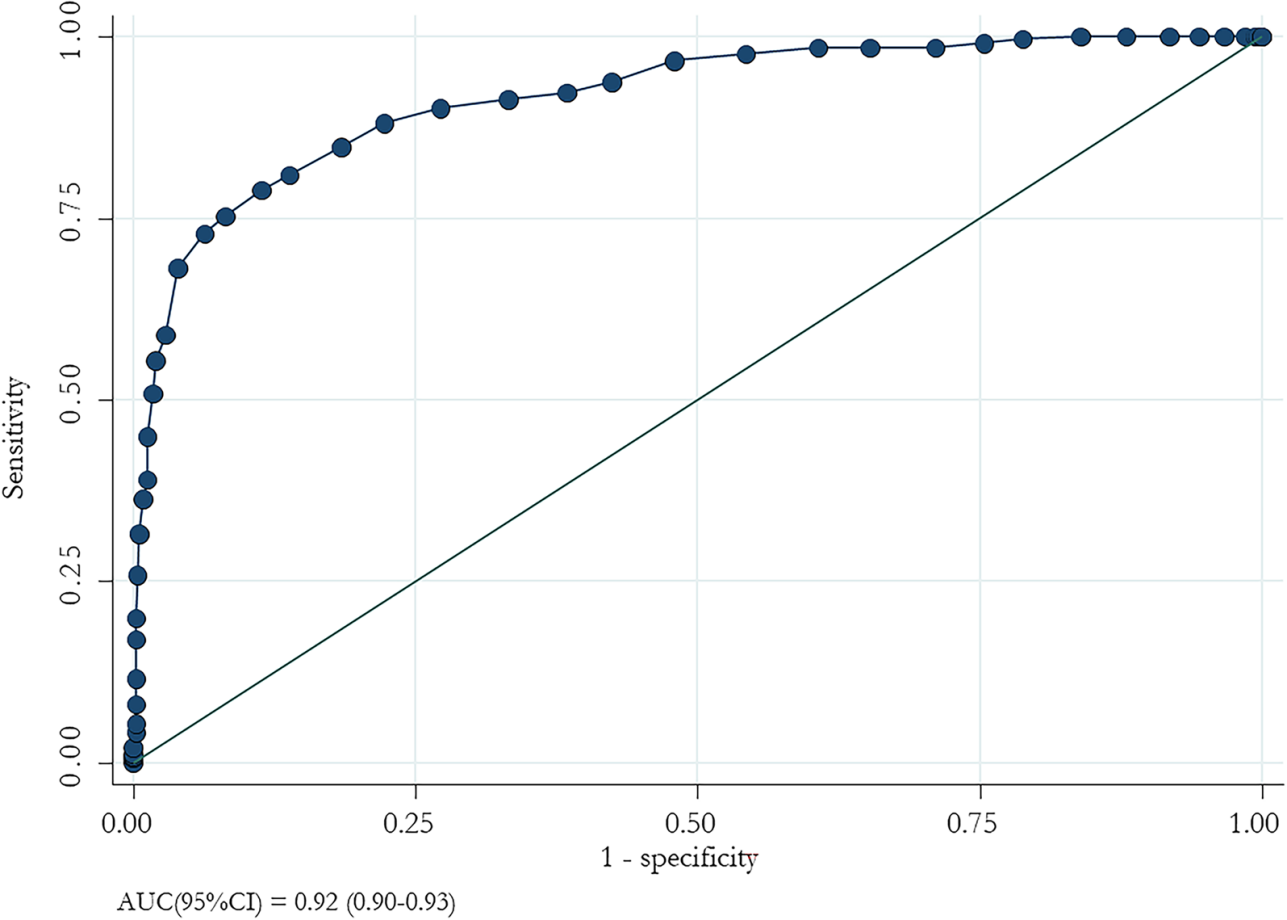
ROC curve analysis of progression risk score

**Fig. 2:**
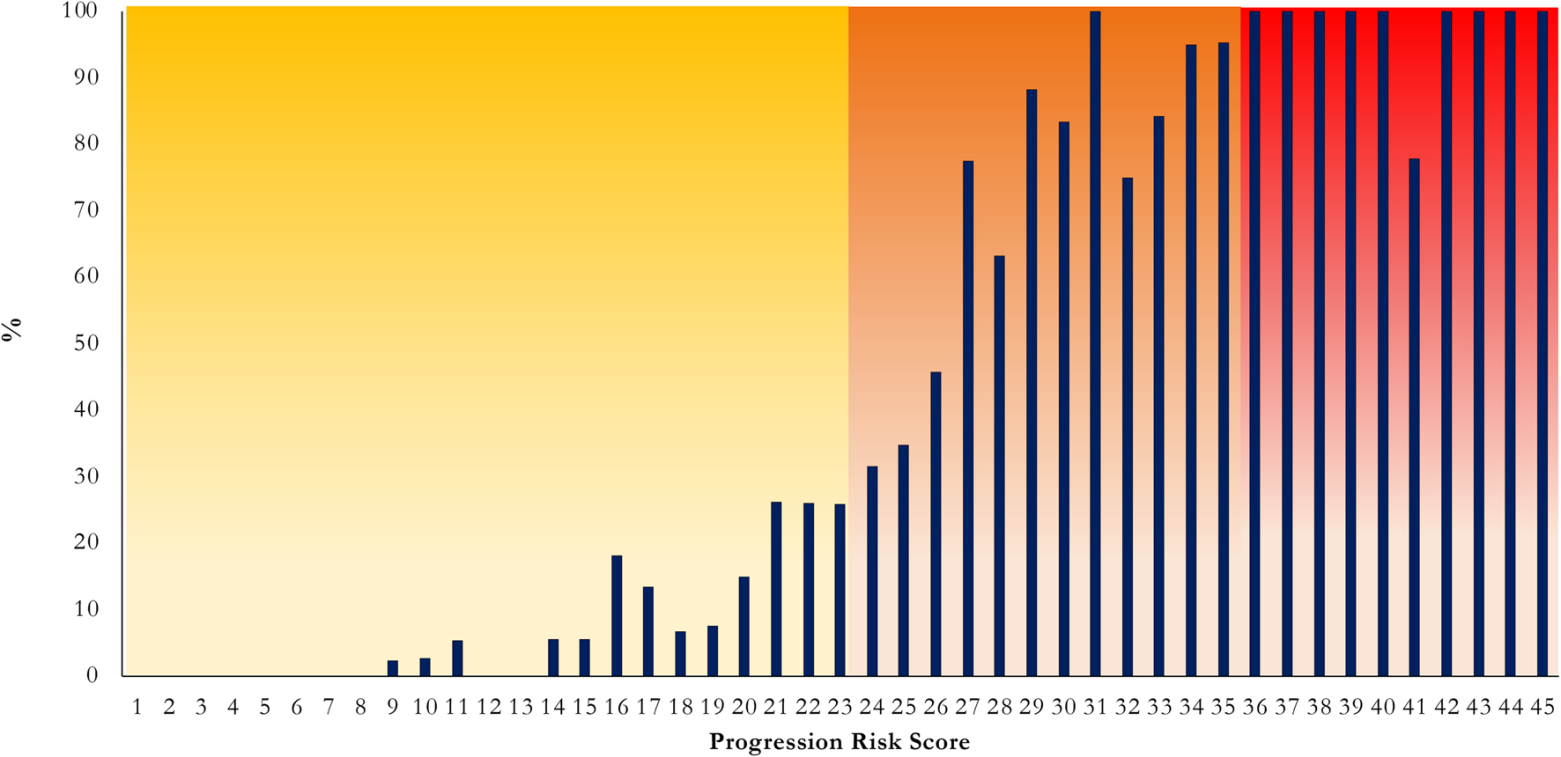
Frequency distribution of progression among different risk score cut points

The ROC curve showed an AUC (95% CI) value of 0.92 (0.90–0.93; Fig. 1). 87.6% were correctly classified with a sensitivity of 72.9% and a specificity 93.7% (Fig. 2).

**Fig. 3:**
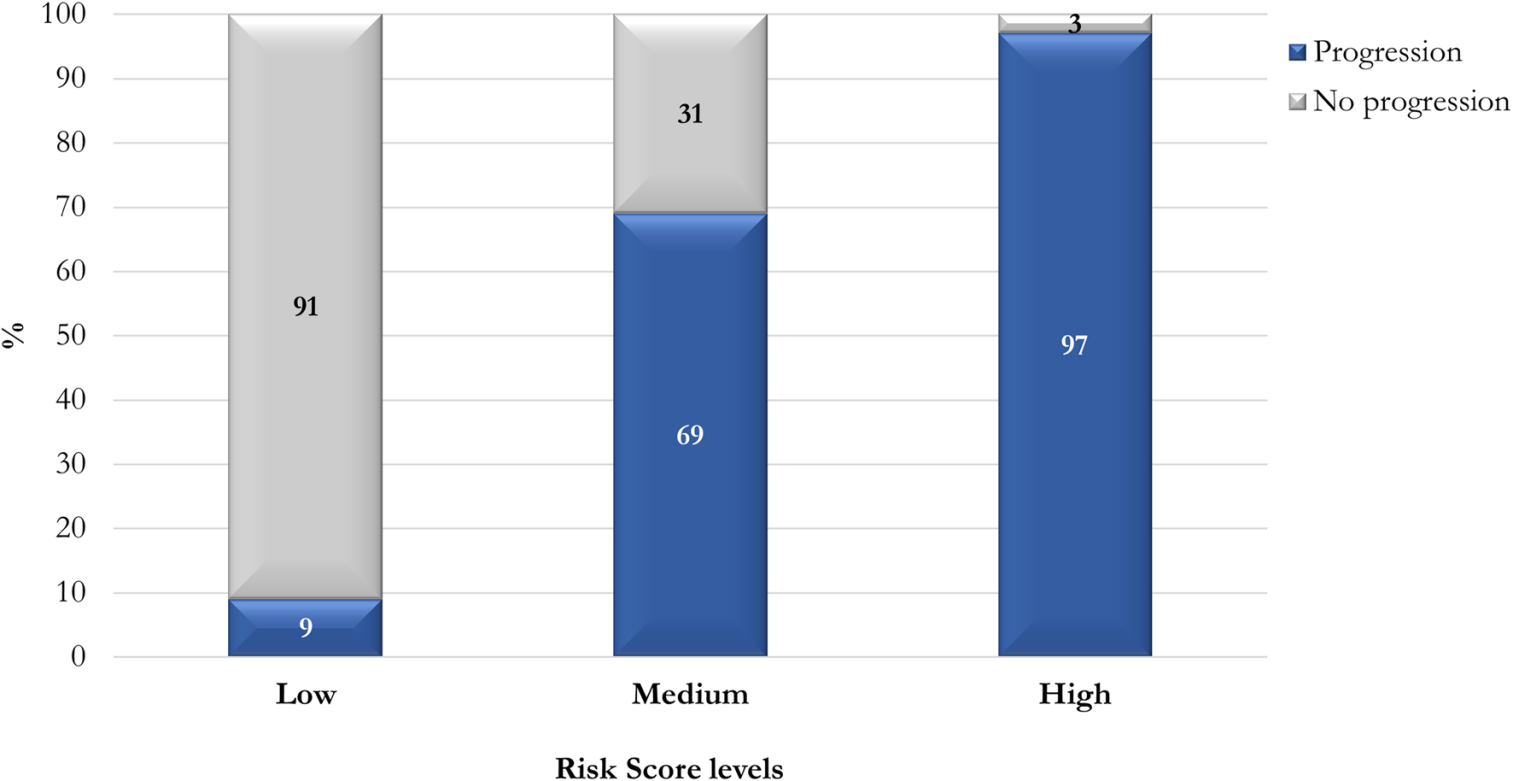
Frequency distribution of progression among different risk score levels

In low-risk group only 9% experienced disease progression, whereas in the medium-risk group the percentage increased to 69%, and to 97% in the high-risk group (Fig. 3).

## Discussion

Our score effectively predicts the progression risk in people with SARS-CoV-2 infection in a pandemic setting with an overworked healthcare system. We identified different variables associated with increased risk.

Authors found that older age is associated with an increased risk of disease progression and death [27–29]. In Italy, only 3.7% of deaths occurred in people under 60 years; the higher risk of death could be explained by the inflammaging, a chronic upregulation of pro-inflammatory status associated with immunosenescence [29].

Our study found that COPD, dementia, hematological tumours, and heart failure were associated with an increased risk of disease severity.

It is well-established that individuals with COPD are at increased risk of developing severe COVID-19 due to chronic lung damage [30, 31]. Numerous studies have investigated this specific population, consistently demonstrating a worse prognosis in terms of severity, complications, and mortality when compared with the general population, confirmed by a meta-analysis which found that the risk of developing COVID-19 was 3.8 times higher among individuals with COPD than those without [30].

However, studies on the role of dementia in COVID-19 are poor. Bianchetti et al. found that among 627 people admitted to a COVID-19 ward, 13.1% had dementia, and 62.2% of them died versus 26.2% without dementia [32]. Additionally, a retrospective study by Harrison et al. found that dementia was a risk factor for increased mortality, potentially explained by alterations of the neuroendocrine and immune systems, as well as by their high frailty index [32].

In our study, hematological malignancy did not reach statistical significance in the univariate analysis, but it was included in the multivariate analysis due to solid clinical evidence [33, 34]. Passamonti et al. reported that among 536 patients with COVID-19 and hematological malignancy, 36.9% died, which is 4 times higher than the general Italian population [33]. Moreover, individuals with hematological malignancy exhibited a lower response to SARS-CoV-2 vaccination. In our study, hematological malignancy was found to increase the risk of disease progression (OR 3.01).

Moreover, chronic heart failure was found to increase disease progression in our study. In another study, individuals with chronic heart failure had a higher percentage of developing severe disease (62% vs. 36.9%) and a 4.6-fold risk of death [35].

Numerous studies on vaccine efficacy found that vaccinated individuals had a significantly lower risk of disease progression (risk ratio 0.38) and COVID-19-related death (risk ratio 0.16) [36].

Ferritin was associated with severity and death due to COVID [37, 38]. It increases in several inflammatory conditions and has an important immunomodulatory effect on mortality and inflammatory processes. The De Ritis ratio (AST/ALT) was also associated with an increased risk of death, although cut-off values were not established [39, 40]. LDH, an enzyme present in the cytosol of all nucleated cells that catalyzes the final step of glycolysis, is a highly sensitive but nonspecific marker of tissue damage due to its distribution [41, 42]. Regarding the WBC, an increase number could be an expression of elevated WBC counts may be indicative of a heightened inflammatory response, which is often associated with severe disease manifestations in COVID-19. This hyperactive immune response can lead to complications such as cytokine storm, contributing to the deterioration of clinical conditions and increasing the risk of severe outcomes [7–10]. Elevated procalcitonin levels in COVID-19 patients are a significant indicator of bacterial co-infection, as procalcitonin is a biomarker that rises in response to bacterial infections. This concomitant bacterial infection can exacerbate the severity of COVID-19 by amplifying the body's inflammatory response and potentially leading to more severe respiratory and systemic complications.

About D-dimer, elevated levels are often associated with an increased risk of thrombotic events, which are common complications in severe COVID-19 cases. High D-Dimer levels can indicate a hypercoagulable state, potentially leading to complications such as deep vein thrombosis or pulmonary embolism, thereby increasing the risk of severe disease progression.

Administration of antivirals and monoclonal antibodies can significantly predict the progression to severe disease. When the study was carried out, the available drugs were monlupiravir, nirmatrelvir/ritonavir, remdesivir (3-day course), casirivimab/imdevimab, and sotrovimab.

Our scoring system was developed when vaccines and early antiviral therapies were available. In the context of predicting COVID-19 progression, Ji et al. described the CALL score model, which includes age, comorbidities, lymphopenia, and LDH and requires only basic laboratory tests. However, it was based on a poor sample size and defined progression based on chest radiological findings rather than the need for oxygen therapy [43]. Gong et al. developed a prognostic nomogram for patients at risk of severe COVID-19 based on age, LDH, CRP, direct bilirubin, red blood cell distribution width, urea, and albumin. However, this complex model does not account for radiological findings [44]. Lee et al. published the KDDH score, which considers age, CRP, LDH, and hemoglobin, and defines progression as the need for oxygen therapy. However, this model does not include radiological signs [45].

The main limitations of those scores are that they did not consider symptoms, such as fever and dyspnea, and were developed using data collected in 2020 when all patients were infected with the wild-type SARS-CoV-2, and no vaccines or preventive therapies were available. Although they show a good sensitivity and specificity, it is unlikely that they could be currently adopted with different variants and multiple treatment options available.

The strength of the present study relies on its high specificity and inclusion of previous vaccination and the use of early antiviral treatment, whereas the limitations are its retrospective and monocenter design, incomplete data on the type of vaccine, unavailability of tixagevimab/cilgavimab, and the lack of data on SARS-CoV-2 previous infections.

## Conclusion

The score showed high specificity, and the risk of underestimating patients' clinical status is low. The main risk factor for disease progression is not having received antiviral or monoclonal antibodies, underscoring that early treatment is fundamental to prevent the disease progression.

In conclusion, this score could significantly affect clinical practice and support clinical decisions at hospital admission.

## Data Availability

All data will be available upon specific request to the authors.
